# Kinetic gating mechanism of DNA damage recognition by Rad4/XPC

**DOI:** 10.1038/ncomms6849

**Published:** 2015-01-06

**Authors:** Xuejing Chen, Yogambigai Velmurugu, Guanqun Zheng, Beomseok Park, Yoonjung Shim, Youngchang Kim, Lili Liu, Bennett Van Houten, Chuan He, Anjum Ansari, Jung-Hyun Min

**Affiliations:** 1 Department of Chemistry, University of Illinois at Chicago, 845 W. Taylor Street, Chicago, Illinois 60607, USA; 2 Department of Physics, University of Illinois at Chicago, 845 W. Taylor Street, Chicago, Illinois 60607, USA; 3 Department of Chemistry, Institute for Biophysical Dynamics, The University of Chicago, 929 E. 57th Street, Chicago, Illinois 60637, USA; 4 Structural Biology Center, Biosciences Division, Argonne National Laboratory, 9700 S. Cass Avenue, Argonne, Illinois 60439, USA; 5 Department of Pharmacology and Chemical Biology, University of Pittsburgh School of Medicine and University of Pittsburgh Cancer Institute, University of Pittsburgh, 5117 Centre Avenue, Pittsburgh, Pennsylvania 15213, USA; 6 Department of Bioengineering, University of Illinois at Chicago, 845 W. Taylor Street, Chicago, Illinois 60607, USA

## Abstract

The xeroderma pigmentosum C (XPC) complex initiates nucleotide excision repair by recognizing DNA lesions before recruiting downstream factors. How XPC detects structurally diverse lesions embedded within normal DNA is unknown. Here we present a crystal structure that captures the yeast XPC orthologue (Rad4) on a single register of undamaged DNA. The structure shows that a disulphide-tethered Rad4 flips out normal nucleotides and adopts a conformation similar to that seen with damaged DNA. Contrary to many DNA repair enzymes that can directly reject non-target sites as structural misfits, our results suggest that Rad4/XPC uses a kinetic gating mechanism whereby lesion selectivity arises from the kinetic competition between DNA opening and the residence time of Rad4/XPC per site. This mechanism is further supported by measurements of Rad4-induced lesion-opening times using temperature-jump perturbation spectroscopy. Kinetic gating may be a general mechanism used by site-specific DNA-binding proteins to minimize time-consuming interrogations of non-target sites.

## Abstract (web summary)

XPC nucleotide excision repair factor is key to starting the repair of diverse helix-distorting DNA lesions caused by environmental insults. Here, the authors propose a kinetic gating mechanism whereby XPC recognizes DNA lesions by preferentially opening damaged sites while readily diffusing away from undamaged sites.

## Graphical abstract

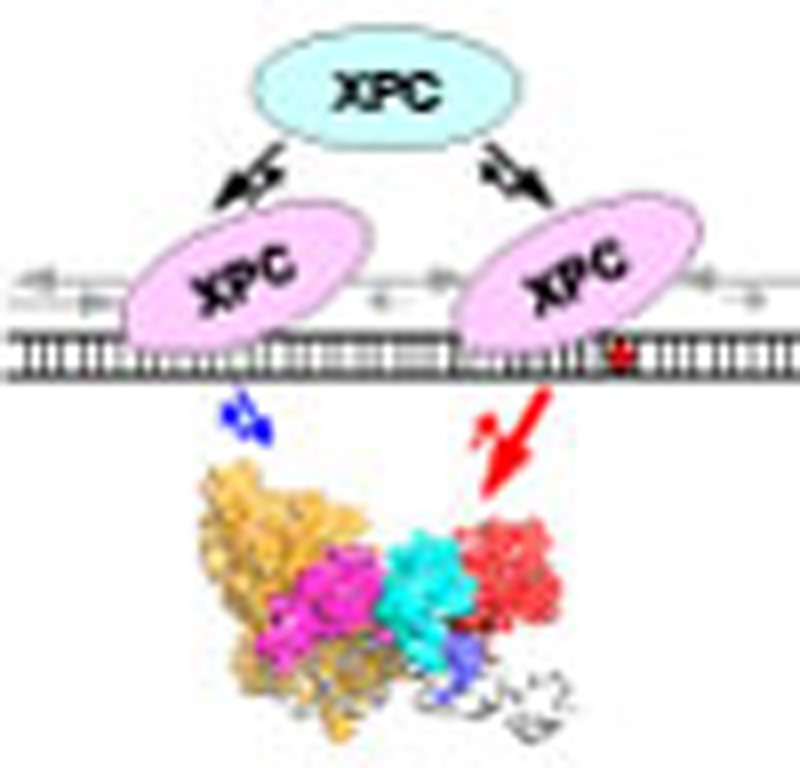

Control and maintenance of the genome is initiated by proteins that recognize and assemble at specific target sites on DNA. These proteins can single out a small number of target sites from a vast excess of closely related non-target sites. How proteins solve this so-called needle-in-the-haystack problem remains a central question in biology^1^. The mammalian global genome nucleotide excision repair (NER) factor, xeroderma pigmentosum C (XPC)–RAD23B complex (hereafter XPC), in particular, faces a difficult challenge. NER repairs diverse helix-distorting/destabilizing DNA damage caused by environmental insults^2^. Impaired NER leads to extreme sun sensitivity and skin cancer predisposition^2^. To initiate NER, XPC needs to recognize an extraordinarily wide variety of DNA lesions based on thermal fluctuations alone. Once bound to a lesion, XPC recruits the multi-subunit transcription factor IIH (TFIIH) that verifies the lesion^3^, which ultimately coordinates the assembly of NER factors, causing excision of a lesion-containing single-stranded DNA and repair synthesis that restores the duplex^2^. Although the lesion-recognition step by XPC is considered the rate-limiting step of NER^4^,^5^, much remains unknown as to how XPC achieves its specific binding efficiently and reliably.

DNA lesions that XPC recognizes include a wide variety of intra-strand crosslinks and helix-distorting adducts, which are formed by ultraviolet light (UV), air and water pollutants, and toxins^2^. XPC efficiently localizes to and recognizes these lesions despite its high affinity for undamaged DNA as measured by *in vitro* assays^6^,^7^,^8^. Notably, XPC resides in DNA-rich chromatin in undamaged cells^9^, which is unique among other mammalian NER proteins^10^,^11^. *In vitro*, XPC can also recognize artificially destabilized DNA such as 2- to 3-base-pair (bp) mismatch bubble (refs 8, 12 and this study), although such mismatches fail the verification step by TFIIH and thus are not excised by NER.

The previous crystal structures of the yeast XPC orthologue, Rad4, bound to model lesions have shown that Rad4 and, by analogy, XPC recognize the damaged DNA site by inserting a β-hairpin into DNA duplex and flipping out damage-containing nucleotide pairs to form an ‘open’ conformation^8^. In this conformation, Rad4 interacts exclusively with the two nucleotides on the undamaged strand without making specific contacts with the two damaged residues of the model UV lesion. This indirect recognition mode^8^,^13^ explained how Rad4/XPC is capable of binding to a variety of lesions. However, it also posed a paradox regarding the mechanism of lesion recognition since the protein can no longer ‘see’ the difference between damaged versus undamaged DNA once the ‘open’ conformation is formed. It has thus been posited that Rad4/XPC may be limited in its ability to fully ‘open’ undamaged DNA in the same way as it opens damaged DNA^8^. To further explore this hypothesis, we sought to solve a crystal structure of the Rad4–Rad23 complex (hereafter referred to as Rad4) bound to undamaged DNA.

Here we present the first crystal structure in which Rad4 is captured on a single register of undamaged DNA using disulphide tethering. Our structure shows that tethered Rad4 flips out normal nucleotide pairs and forms the same ‘open’ conformation as with damaged DNA. These results indicate that structural discrimination between normal and damaged DNA cannot be the basis for lesion recognition by Rad4/XPC, and instead points to a novel ‘kinetic gating’ mechanism, whereby the DNA lesion selectivity arises mainly from the kinetic competition between Rad4-induced DNA opening and the residence time of Rad4 at a given site. While prolonging residence time, for example, by tethering, induces full opening (‘recognition’) of even normal DNA, we propose that freely diffusing Rad4 has a lower probability to open undamaged DNA than damaged DNA. This mechanism is further supported by direct measurements of Rad4-induced opening times (~7 ms) at a helix-destabilizing lesion, using temperature-jump perturbation spectroscopy. The opening time for stable, undamaged DNA is expected to be orders of magnitude slower than that for opening an unstable lesion, and also significantly longer than the residence time of Rad4/XPC at the site, providing keys to lesion discrimination. This ‘kinetic gating’ provides novel insights into Rad4/XPC functions within and beyond NER^14^,^15^,^16^,^17^,^18^, and it may be a general mechanism for site-specific DNA-binding proteins to facilitate target search and recognition in the genome.

## Results

### Crystal structure of Rad4–Rad23 tethered to undamaged DNA

**Figure 1:**
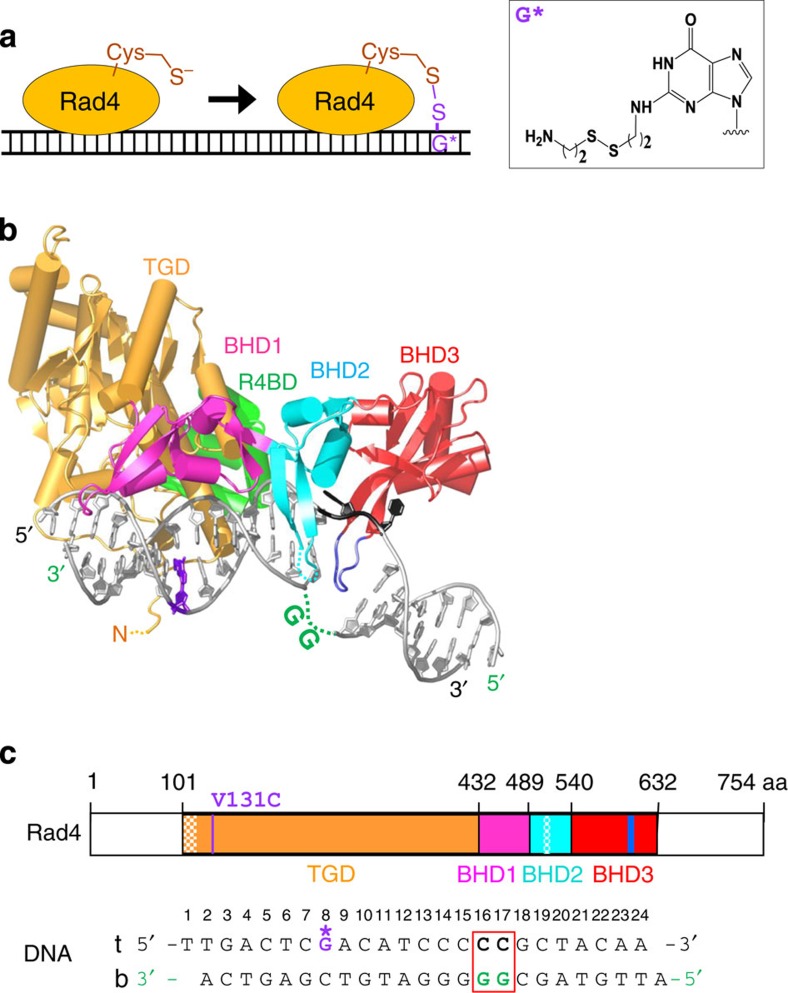
Crystal structure determination of Rad4–Rad23 bound to undamaged DNA using disulphide crosslinking. (**a**) Crosslinking scheme to tether Rad4 on DNA. A disulphide crosslink between cysteine 131 in Rad4 and a modified nucleotide (G*) on DNA was introduced to capture the protein–DNA complex in a defined register of binding. The chemical structure of G* is shown on the right. (**b**) Structure of Rad4–Rad23 tethered to undamaged DNA (PDB code 4U29). The transglutaminase domain (TGD, orange) and β-hairpin domain 1 (BHD1, magenta) of Rad4 bind to an 11-bp duplex segment of the DNA (silver), while BHD2 (cyan) and BHD3 (red) of Rad4 bind to a 4-bp segment in which two nucleotide pairs are flipped out. The tip of the long β-hairpin in BHD3 (residues 599–605, blue) is inserted into the DNA duplex and fills the gap created by the flipped-out nucleotides. The crosslinked, G*8 in DNA and Cys131 in TGD are in purple. The two flipped-out cytidines (black) are bound by BHD2-BHD3, while the complementary guanosines flipped out away from the protein are disordered in the crystal (green dotted line). Rad23 binds to TGD through its Rad4-binding domain (R4BD, light green). ‘N’ indicates the N terminus of Rad4. (**c**; top) Domain arrangements and boundaries of Rad4 used in this study. The colour scheme is the same as in **b**. The crystallized Rad4 construct spans residues 101–632 as before^8^ and contains point mutations V131C (purple) and C132S. These residues are at the end of the N terminus of TGD, away from the BHD2-BHD3 bound to flipped-out nucleotides. The disordered regions in the crystal are checkered. Rad23 construct is the same as in ref. 8. (Bottom) DNA duplex sequence used for the structure determination. The nucleotide pairs flipped out by Rad4 are boxed in red. The disulphide-modified nucleotide, G*8, was at a position 8-bp away from the flipped-out ones (CC/GG at 16 and 17). Top strand (‘t’) corresponds to the undamaged strand and the bottom (‘b’) to the damaged strand in the lesion-bound structure^8^ (PDB code 2QSG).

**Figure 2:**
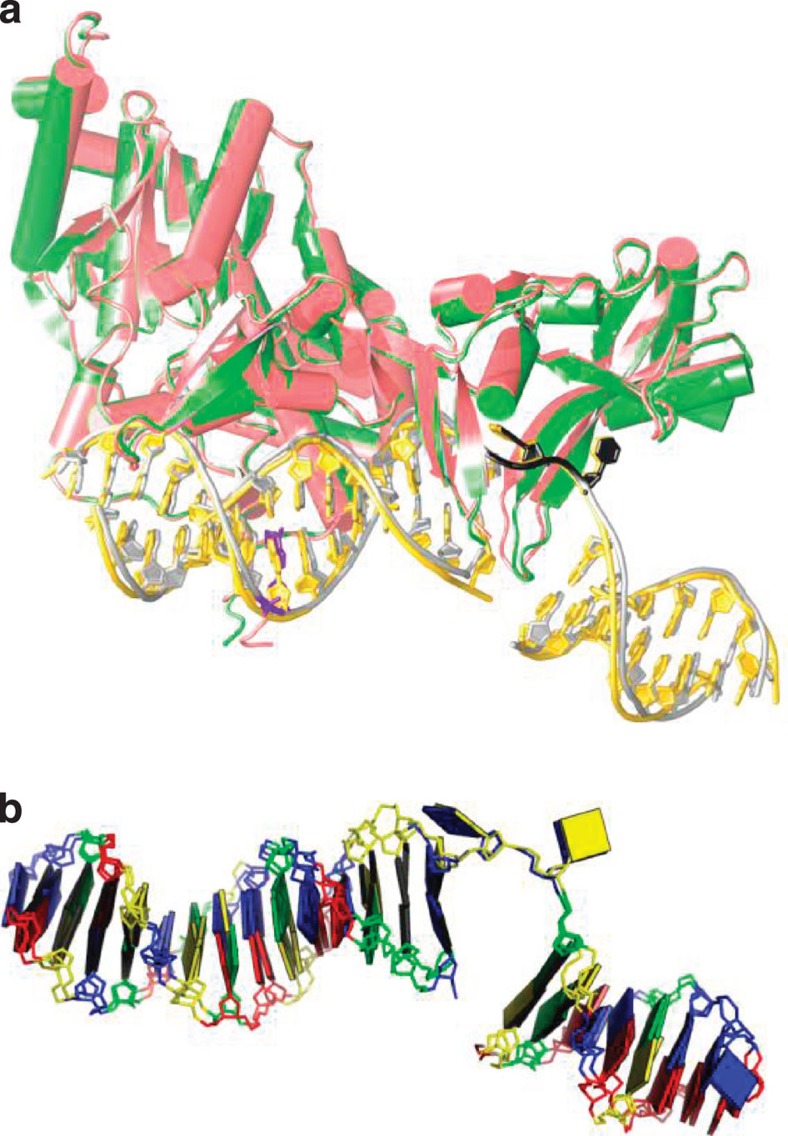
Tethered Rad4 opens up undamaged DNA nucleotide pairs as it does to damaged ones. (**a**) Superposition of the Rad4–undamaged DNA (green-silver, this study) and the Rad4-damaged DNA^8^ (red-gold, PDB code 2QSH) structures. The crosslinked G* in DNA and Cys131 in Rad4 are indicated in purple and the flipped-out nucleotides are indicated in black in the undamaged DNA-bound structure. (**b**) Rad4-bound DNA conformations are nearly identical between the damaged- and the undamaged-bound conformations. Cytidines are represented in yellow boxes, thymidines in blue, guanosines in green and adenosines in red. The figure is generated by 3DNA^44^.

Obtaining diffracting crystals of the Rad4–undamaged DNA complex was initially challenging, presumably because Rad4 can bind in multiple different registers or binding sites on undamaged DNA. To overcome this problem, we covalently tethered Rad4 to undamaged DNA in a fixed register using a disulphide crosslinking method^19^ and solved a 3.05 Å structure of this complex (Fig. 1; Supplementary Figs 1 and 2; Table 1). To our surprise, we found that this tethered complex formed an ‘open’ conformation that is essentially indistinguishable from the lesion-bound structure^8^ (Fig. 2). Note that the disulphide tethering was made at a position distant from the flipped-out nucleotides (Fig. 1b,c) and, therefore, it is unlikely that the ‘open’ conformation was due to structural perturbations from crosslinking. Consistent with this notion, the electrophoretic mobility shift assays show that the modifications made in the crystallized construct do not affect the damage-specific binding of Rad4 compared with the unmodified, full-length protein (Supplementary Fig. 1). The overall root mean squared deviation^20^ between this complex and the lesion-bound complex structures is 0.99 Å for 2,388 main-chain atoms including both protein and DNA. The distance between the crosslinked residues (Cα of V131 in Rad4 and C2 of G*8 in the top strand of the DNA; Fig. 1c) is 8.8 Å in this structure, only 0.3 Å shorter than the distance between equivalent atoms (9.1 Å between Cα of C131 in Rad4 and C2 of A8 in the top strand of the DNA) in the lesion-bound structure (Protein Data Bank (PDB) code 2QSH)^8^, further confirming that the crosslinking did not induce structural distortions in the protein–DNA complex.

### Atomic force microscopy shows that Rad4 bends undamaged DNA

**Figure 3:**
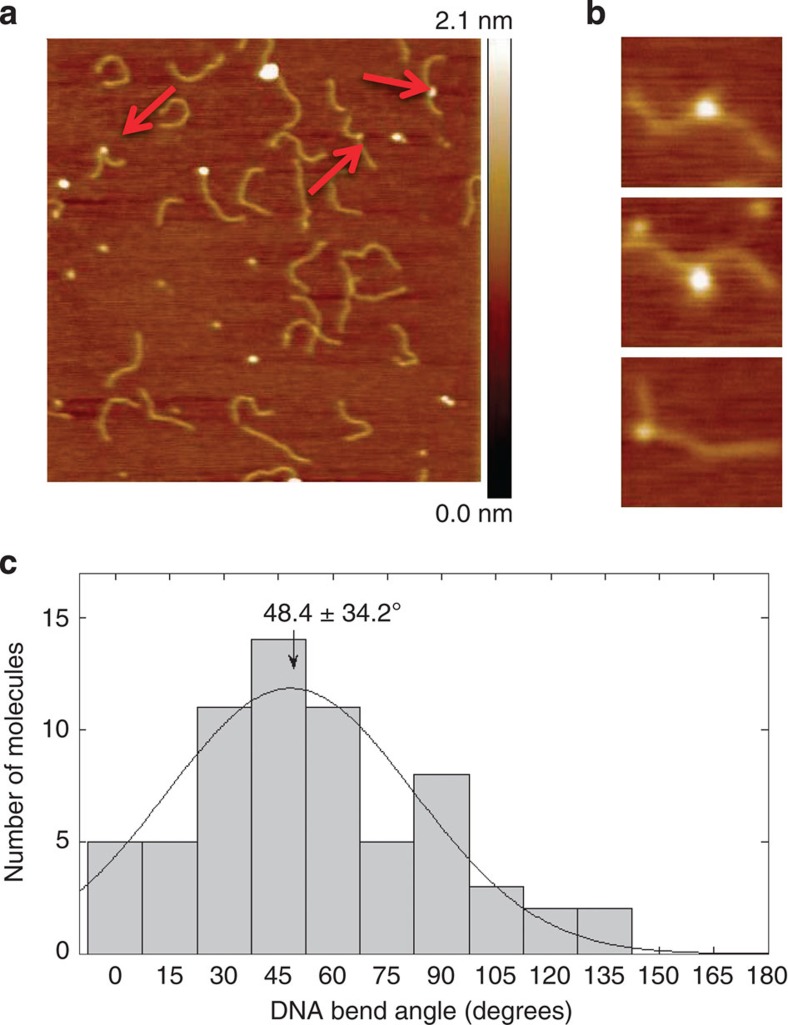
AFM on Rad4 binding to 514-bp undamaged DNA. (**a**) AFM image of Rad4 with 514-bp undamaged DNA. The arrows indicate proteins bound on DNA. Scan area is 1 μm × 1 μm. (**b**) Zoomed images of examples of Rad4 bound on DNA. (**c**) Histogram of bend angle distribution for Rad4 bound to undamaged DNA. Total of 67 molecules were measured from three independent depositions. The solid-line curve represents fitting of these data to a Gaussian distribution in MATLAB, providing the centre of bend angle as 48.4° (±34.2°). The uncertainty in the bend angle is the sample s.d. derived from the Gaussian fit.

In this Rad4–undamaged DNA structure, the DNA is bent by ~42° as in the lesion-bound structure. Consistent with this, an atomic force microscopy (AFM) study of Rad4 bound (but not tethered) to a 514-bp undamaged DNA also showed that the DNA is bent (48.4±34.2°; Fig. 3). These results agree remarkably with a previous AFM study of human XPC bound to undamaged DNA segments (49°±36°; ref. 21). We also observed that a significant fraction of Rad4 binds to the ends of the DNA (Supplementary Fig. 3a). Only the fraction that was bound internally was used for analysis of the DNA bending angles (Supplementary Fig. 3b).

### Nonspecific Rad4–DNA structure suggests kinetic read-out

To our knowledge, the presented crystal structure is the first high-resolution structure of a nonspecific DNA-bound, DNA repair protein that relies exclusively on indirect read-out and makes no direct contacts with the damaged nucleotides in the specific recognition complex. Our finding that a nonspecific complex is structurally identical to the specific complex was unexpected in light of previous studies on multiple other proteins that had shown clear differences between the two structures^22^,^23^,^24^,^25^,^26^,^27^, including those that used similar crosslinking approaches^24^,^25^,^26^. These earlier studies have been on sequence-specific DNA-binding proteins or damage-repair enzymes. For these proteins, target recognition lies, at least in part, in the ability of the proteins to directly accommodate target DNA sites and reject non-target sites as structural misfits. In contrast, our results reveal that such direct structural distinction cannot be the basis for the lesion recognition by Rad4. Instead, these results suggest that the key to Rad4’s recognition mechanism lies in the differential probability that Rad4 can open up a damaged versus undamaged DNA site before it diffuses away.

### Rad4-induced DNA-opening rates measured by temperature jump

A key determinant of the ‘opening’ probability is the free energy barrier encountered by Rad4 to fully open DNA to form the recognition complex, which, in turn, determines the Rad4-induced opening rate. Here we have measured these opening rates using temperature-jump (T-jump) spectroscopy. Briefly, the Rad4 construct identical to the one used in crystallography studies^8^ was bound to model lesions (2- or 3-bp mismatch bubbles in two different sequence contexts) in which 2-aminopurine (2AP, an adenine analogue) was incorporated as a fluorescent probe sensitive to DNA-opening events^28^ (see Supplementary Table 1 for DNA sequences). All equilibrium and kinetics measurements reported here were carried out on untethered Rad4–DNA complexes.

**Figure 4:**
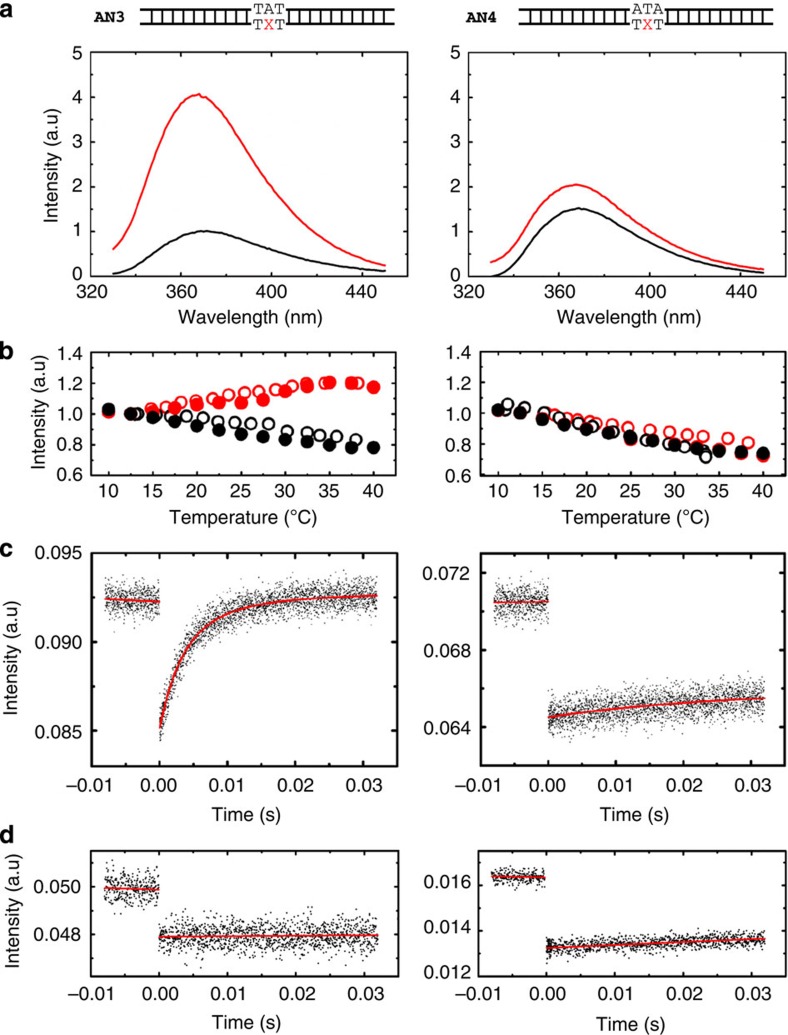
Rad4-induced DNA-opening dynamics measured by T-jump spectroscopy using 2AP fluorescence as a probe. (**a**) 2AP (X in the schematic representation of DNA substrates) was placed within 3-bp mismatch DNA (AN3) and matched DNA (AN4; Supplementary Table 1). The 2AP fluorescence emission spectra were measured for DNA alone (black) and Rad4–DNA complexes (red) with excitation at 314 nm at 25 °C (*left*: AN3; *right*: AN4). The 2AP fluorescence emission intensities increase 4.0 (±0.7)-fold and 1.4 (±0.1)-fold on Rad4 binding to mismatch and matched DNA, respectively. All measurements were done with untethered Rad4. Protein and DNA concentrations were 10 μM each. (**b**) The maxima of the equilibrium 2AP fluorescence emission, measured at 365 nm, are plotted as a function of temperature for DNA alone (black) and Rad4–DNA complexes (red; *left*: AN3; *right*: AN4). Open and filled symbols are for two independent sets of measurements on each sample. In each panel, the intensities for free DNA and DNA in complex have been normalized to match at the lowest temperature. (**c**) Relaxation kinetic traces measured in response to a ~7 °C T-jump show (*left*) single-exponential kinetics, with relaxation time 5.1±0.5 ms (at final temperature 26 °C) for Rad4–mismatch DNA, and (*right*) much slower kinetics, with relaxation time 190±42 ms (at final temperature 29 °C) for Rad4–matched DNA. The uncertainties in the relaxation times are sample s.d. from two sets of measurements. Note that the *K*_d_ values of Rad4 bound to mismatch or matched DNA are in nanomolar range (Supplementary Fig. 1), well below the 60 μM concentrations used in the T-jump experiments. (**d**) Relaxation kinetics measured on the DNA-only samples: AN3 alone, in response to a 6 °C T-jump (*left*), and AN4 alone, in response to a 10 °C T-jump (*right*), exhibit much slower kinetics, with relaxation times 341±70 and 240±35 ms, respectively, consistent with the T-jump recovery kinetics (see also Supplementary Fig. 4d). The relaxation times for samples that exhibit only the slow kinetics were determined by making measurements over a longer time window, up to 80–320 ms.

Specific binding of Rad4 to the mismatch model lesions resulted in a greater than fourfold increase in 2AP fluorescence of the DNA at equilibrium (Fig. 4a, *left*; Supplementary Fig. 4a). While small (less than approximately twofold) increase in 2AP fluorescence can come from unstacking of 2AP from its neighbours, for example, from partial duplex melting or unwinding, we attribute the much larger increase in the lesion-bound, specific complexes as arising from Rad4-induced DNA ‘opening’ that results in the complete flipping out of two nucleotide pairs as seen in the crystal structures^8^. A temperature scan from 10 to 40 °C showed that 2AP fluorescence of the specific, Rad4–mismatch DNA complexes increases as temperature is raised (Fig. 4b, *left* red), indicating that the extent of 2AP unstacking and/or the population of fully flipped-out conformations increases at higher temperatures. In contrast, the 2AP fluorescence intensity in mismatch DNA alone (Fig. 4b, *left* black) or in undamaged, matched DNA with or without Rad4 (Fig. 4b, *right*) decreases monotonically with increasing temperature, which reflects a temperature-dependent decrease in 2AP quantum yield.

To monitor conformational relaxation kinetics of Rad4 bound to DNA, samples were rapidly perturbed by a T-jump of ~5–10 °C with a ~10-ns infrared laser pulse, and the temporal response of the 2AP fluorescence was monitored as the ensemble of molecules re-equilibrated to the distribution corresponding to the higher temperature. Our T-jump apparatus can monitor conformational relaxation kinetics of protein–DNA interactions in a time window from ~5 μs to 50 ms, as demonstrated with other DNA-binding proteins^29^. T-jump measurements on the ensemble of Rad4–mismatch DNA complexes revealed relaxation kinetics with time constants of 5.5±0.5 and 7.7±2.0 ms for the 3-bp and 2-bp mismatch constructs, respectively (interpolated at 25°C), indicating that the kinetics were also sequence context independent (Fig. 4c, *left*; Supplementary Fig. 4c). We attribute these to the Rad4-induced DNA ‘opening’ kinetics. Similar relaxation rates were also observed with full-length Rad4 complex on the 3-bp mismatch DNA (Supplementary Fig. 5). In contrast, measurements on Rad4 nonspecifically bound (but not tethered) to undamaged, matched DNA showed a much smaller increase in 2AP fluorescence on Rad4 binding and no relaxation kinetics on the millisecond timescale (Fig. 4, *right*). Instead, the complex showed much slower kinetics on timescales characteristic of the re-equilibration of the heated sample temperature back to the initial (bath) temperature (T-jump recovery), similar to the control samples, matched and mismatch DNA in the absence of Rad4 (Fig. 4d; Supplementary Fig. 4d). The apparent absence of conformational relaxation kinetics on the untethered complex with undamaged DNA was somewhat unexpected in view of the ‘open’ crystal structure of the tethered complex. The absence of kinetics can arise from heterogeneous binding registers (since Rad4-induced opening of DNA at a site some distance away from the fixed 2AP site will not be reported by 2AP fluorescence change) and/or slow relaxation kinetics not resolved in the T-jump time window.

**Figure 5:**
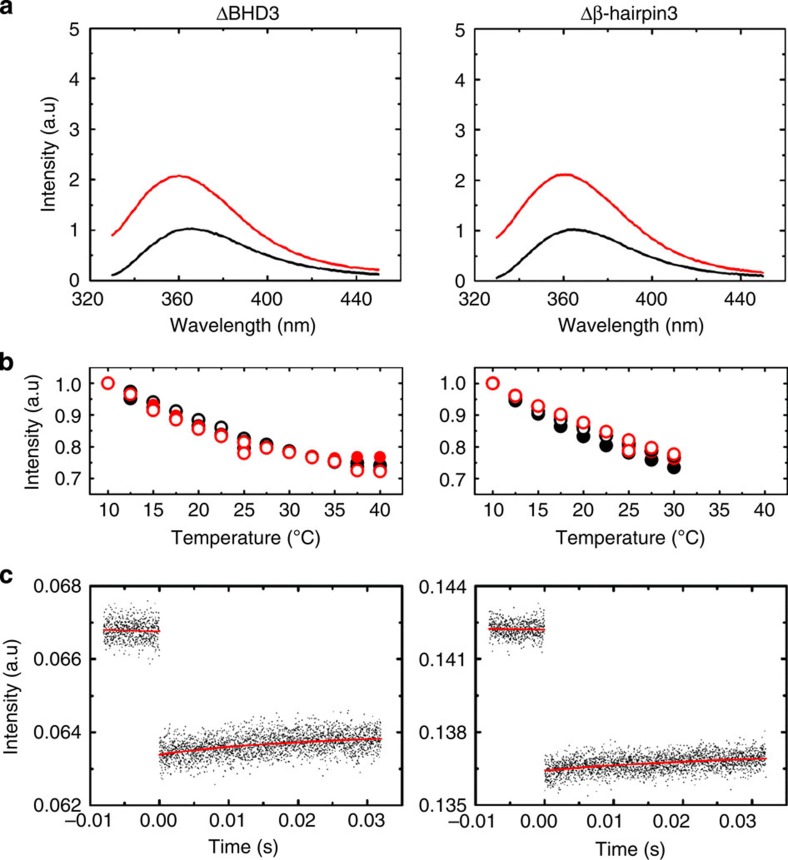
2AP fluorescence and T-jump measurements to examine Rad4-induced DNA-opening dynamics with Rad4 β-hairpin mutants. Equilibrium and relaxation kinetics measurements were carried out on 3-bp mismatch DNA (AN3) bound to two β-hairpin mutants of Rad4, ΔBHD3 (*left*) and Δβ-hairpin3 (*right*). (**a**) The 2AP fluorescence emission spectra measured for DNA alone (black) and Rad4–DNA complexes (red) with excitation at 314 nm at 25 °C. The 2AP fluorescence emission intensities of AN3 DNA increase 2.1 (±0.1)-fold and 2.3 (±0.1)-fold on binding of ΔBHD3 and Δβ-hairpin3, respectively, compared with DNA alone. (**b**) The maxima of the equilibrium 2AP fluorescence emission measured at 365 nm are plotted as a function of temperature for DNA alone (black) and Rad4–DNA complexes (red). The open and filled symbols are for two independent sets of measurements on each sample. The intensities are normalized to match at the lowest temperature. (**c**) T-jump measurements on the β-hairpin mutants of Rad4–DNA complexes do not show any relaxation kinetics other than the slow recovery of the T-jump itself.

Structural studies have shown that the β-hairpin in the β-hairpin domain 3 (BHD3) inserts into the DNA duplex on forming the ‘open’ conformation (Fig. 1; ref. 8); this β-hairpin is also shown to be important in the ability of Rad4/XPC to recognize DNA lesions in biochemical and cellular studies (Supplementary Fig. 1; refs 18, 30). To probe what role the β-hairpin plays in the observed increase in 2AP fluorescence in the mismatch DNA on Rad4 binding, and in the relaxation kinetics when perturbed by T-jump, we have performed corresponding 2AP fluorescence measurements with mutant Rad4 (Fig. 5) that lack either the β-hairpin in BHD3 (Δβ-hairpin3, missing residues 599–605 indicated in blue in Fig. 1b,c) or the entire BHD3 domain (ΔBHD3, missing residues 541–632 indicated in red in Fig. 1b,c). The 2AP fluorescence of the mismatch DNA in complex with either of the Rad4 mutants shows a temperature dependence very similar to that of free DNA (Fig. 5b). These results contrast with the behaviour of the wild-type Rad4–mismatch DNA complex, which showed an increase in fluorescence emission intensity with increasing temperature (Fig. 4b, *left*). Furthermore, no relaxation kinetics (other than the T-jump recovery kinetics) was observed with these β-hairpin mutants (Fig. 5c). The results indicate that the β-hairpin is indeed critical for forming and stabilizing the ‘open’ conformation, consistent with other studies (Supplementary Fig. 1; refs 8, 18).

**Figure 6:**
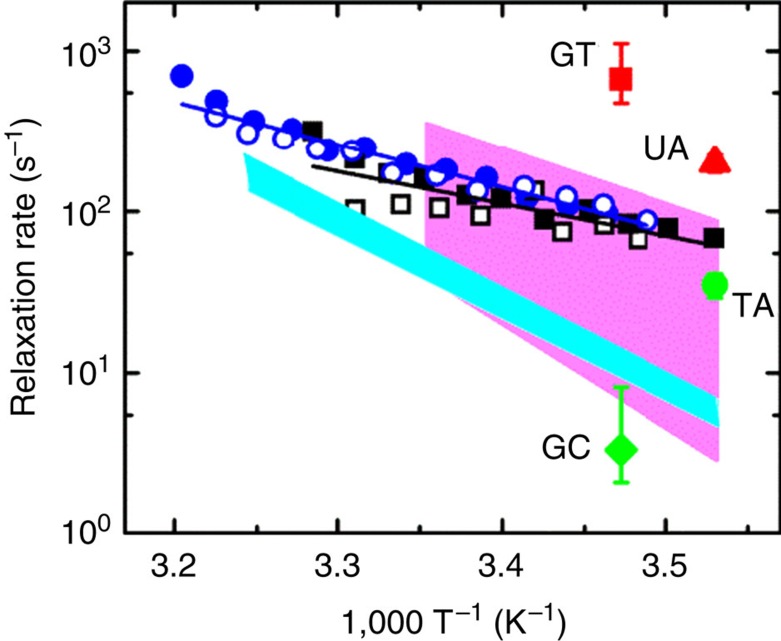
Arrhenius plots of relaxation rates measured on Rad4–mismatch DNA complexes. Relaxation rates obtained from T-jump measurements on Rad4–mismatch DNA (AN3 in blue circles and AN21 in black squares) are plotted versus the inverse of the final temperature after the T-jump. The open and filled symbols are for two independent sets of measurements on each sample. The continuous (blue and black) lines are an Arrhenius fit to the relaxation rates for each sample; the activation enthalpies obtained from the Arrhenius fit are 12.1±2.5 and 7.9±4.7 kcal mol^−1^, respectively, for the two sequences, and the relaxation times interpolated at 25 °C are 5.5±0.5 and 7.7±2.0 ms, respectively. The uncertainties in the activation enthalpies and relaxation times are sample s.d. obtained from two independent sets of measurements. The pink (cyan) shaded regions represent the range of base-pair opening (‘breathing’) rates for A/T (G/C) base pairs, from NMR imino proton exchange measurements of Coman and Russu^45^. The opening rates and their ranges are also indicated for G/T mismatch (red square)^31^, U/A mismatch (red triangle)^23^, T/A base pair (green circle)^23^, and G/C base pair (green diamond)^31^.

The observed Rad4-induced DNA-opening kinetics of ~7 ms on mismatch DNA are almost tenfold slower than the spontaneous base-pair ‘breathing’ measured even for a single (GT) mismatch (Fig. 6; Supplementary Fig. 5)^31^. These results show that the free energy barrier for forming the ‘open’ structure is higher than for the smaller ‘breathing’ motions, and thus indicate that the rate-limiting transition state for ‘opening’ entails extensive structural deformation around the lesion. Notably, NER lesions often weaken base-pair hydrogen-bonding and stacking interactions and render the DNA more deformable with increased propensity for local bending/unwinding/nucleotide flipping^2^. Thus, the free energy barrier for Rad4-induced opening of undamaged DNA is expected to be even higher compared with damaged DNA. A recent simulations study estimated that the free energy barrier for flipping out normal DNA could be 5–8 kcal mol^−1^ higher than for a bulky lesion^32^. Assuming that this entire difference appears in the free energy barrier for Rad4-induced DNA-opening kinetics, the opening times for normal DNA bases could well be >4,000-fold longer than the ~7 ms opening time observed for our model lesion.

**Figure 7:**
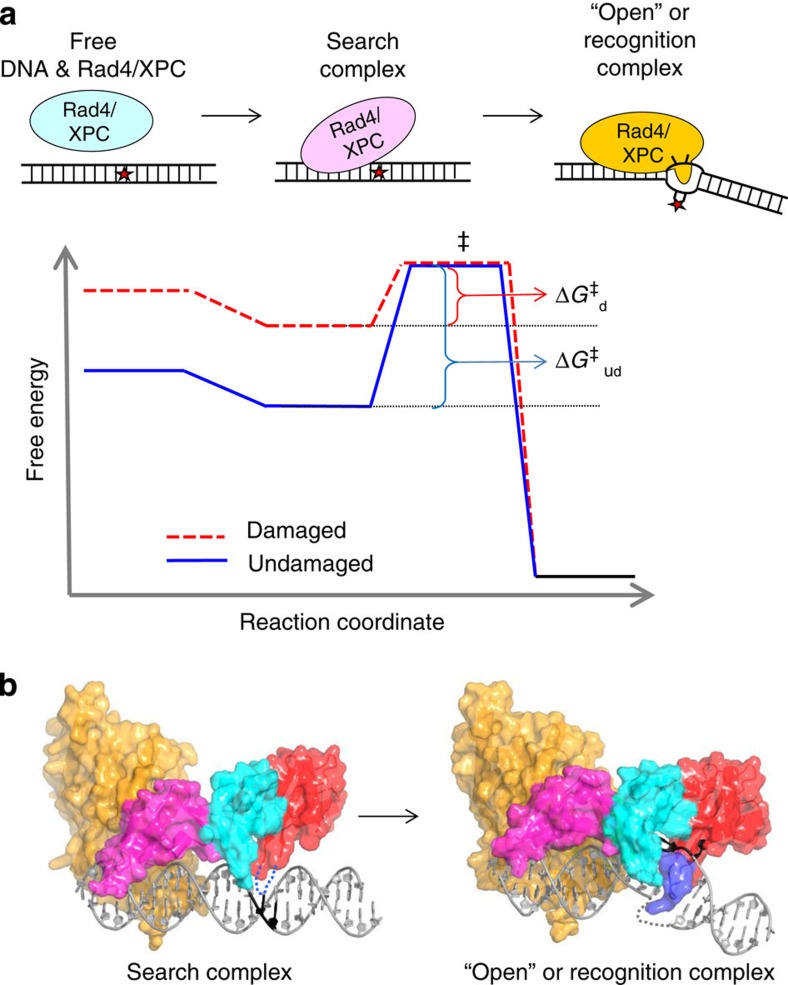
Free energy profile for DNA opening by Rad4/XPC at damaged and undamaged sites. (**a**) A schematic representation of the free energy profile of the Rad4/XPC-induced DNA-opening process is shown: free Rad4/XPC is indicated in cyan, Rad4/XPC in the ‘search’ mode in pink and in the ‘open’ recognition complex in orange. The free energy surface for opening damaged DNA sites is in red and that for undamaged sites is in blue. Δ*G*_d_^‡^ and ΔG^‡^_ud_ denote the free energy barriers to open damaged and undamaged sites in the complex, respectively. (**b**) Structural models for Rad4 in a ‘search’ mode (*left*) and in a recognition complex (*right*). Here we used the apo-Rad4–Rad23 structure (PDB code 2QSF)^8^ to model Rad4 nonspecifically bound to DNA in a search mode. The β-hairpin in BHD3 in this conformation can be flexible (blue dotted line), while the DNA duplex largely retains the B-DNA conformation. On the contrary, the β-hairpin in the recognition complex is inserted into the DNA duplex (blue surface representation) and stabilizes the ‘open’ structure of the DNA. The ‘open’ complex is formed with damaged DNA^8^ and also with undamaged DNA when Rad4’s residence time is prolonged (this study).

Taken together, our present study strongly suggests that the kinetic barriers for forming the ‘open’ conformation are key to distinguishing damaged versus undamaged DNA, although the final ‘open’ structures appear to be nearly the same for both DNA. Figure 7a illustrates this notion. In this schematic, the free energies of the final ‘open’ structures, as well as the corresponding transition states for forming such structures, are assumed to be very similar for the damaged and undamaged DNA. However, the free energies when Rad4/XPC loosely and nonspecifically interacts with the DNA in the ‘search’ mode^33^ are assumed to differ by an amount equal to the free energy difference between undamaged and damaged free DNA. Although an experimental structure of Rad4/XPC in an untethered, bona fide search mode is lacking, one can anticipate that the DNA duplex in this mode remains largely in the conformation of free DNA (as modelled in Fig. 7b). Consequently, the higher free energy barrier for Rad4/XPC ‘opening’ undamaged versus damaged DNA mainly arises from the lower free energy of the Rad4/XPC–normal DNA complex in the search mode that reflects the higher stability of normal DNA than that of damaged DNA.

### Interplay between residence time and opening time

Given a free energy barrier to open a DNA site, the overall probability of Rad4 opening the site also depends on whether this barrier can be overcome by Rad4 before it steps to the next site or falls off, that is, the residence time of Rad4 at the site. The opening probability per site then can be expressed as the ratio of the opening time (*t*_op_) and the residence time (*t*_res_), and this probability increases exponentially as the ratio decreases: *P*_open_=1−exp(−*t*_res_/*t*_op_). In our structural study, chemical tethering of Rad4 on DNA prolonged Rad4’s residence time indefinitely, thus allowing even undamaged DNA to be fully opened. However, under untethered conditions, the protein is free to diffuse away from a given site, thus decreasing the residence time per site. If *t*_res_≪*t*_op_, this opening probability is likely to be small. While there are no direct measurements of the residence time for Rad4/XPC on DNA, several measurements have characterized diffusion of DNA repair proteins on undamaged, nonspecific DNA. These measurements yield a wide range of residence times per base pair, 0.1 μs to 0.3 ms (Supplementary Table 2)^34^,^35^,^36^, all of which are significantly shorter than the Rad4-induced opening time expected for undamaged DNA (>>7 ms). This estimate for opening normal DNA is based on the 7 ms opening time that we measured for mismatch DNA at 25 °C (Fig. 6) and the simulations study^32^ that suggests significantly larger free energy barrier for flipping out normal bases in matched DNA (as illustrated in Fig. 7). Assuming that the residence time of Rad4 on undamaged DNA falls in the submillisecond range, the probability that Rad4 will open a normal site is expected to be exceedingly small. Although it remains to be determined, we speculate that the presence of an NER lesion that distorts or destabilizes DNA not only decreases the opening time, but may also increase the residence time of Rad4/XPC on the lesion compared with an undamaged site, further contributing to the selective opening and thus recognition of NER lesions. On the other hand, certain carcinogen adducts resistant to NER^2^,^37^ may evade detection in part because they present very high free energy barriers for being opened by Rad4 compared with its residence time, and thus their opening probability may be similar to or worse than that of undamaged DNA.

## Discussion

**Figure 8:**
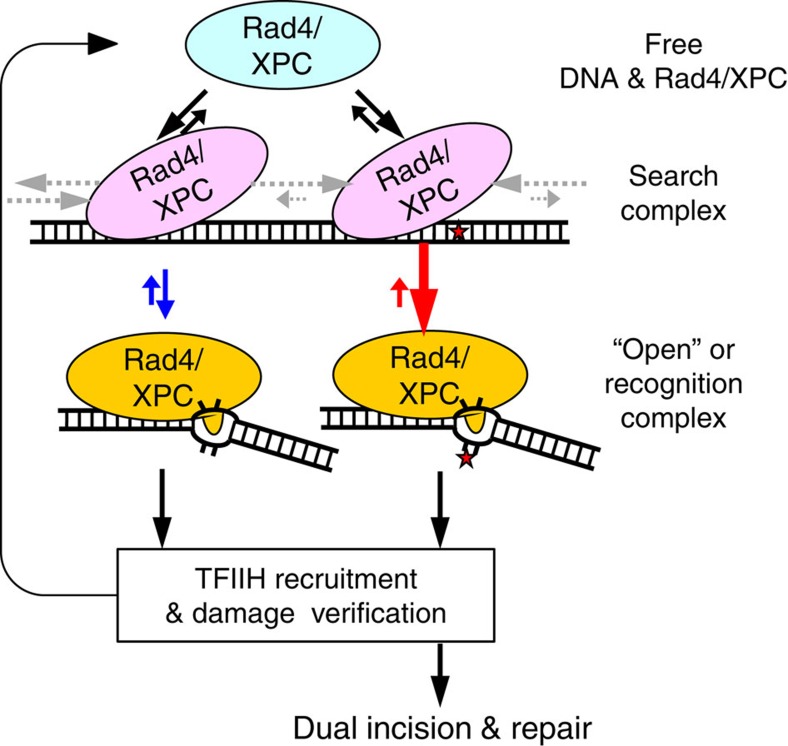
‘Kinetic gating’ mechanism for DNA damage recognition by Rad4/XPC. This schematic illustrates that the lesion recognition by Rad4/XPC is controlled by the competition between Rad4’s residence time per site when it is in the ‘search’ mode (pink) and its opening time to form the ‘open’ or recognition complex (orange). The diffusion rates that control the residence time are indicated by the grey arrows. The opening (and its reverse) rates are depicted in red and blue arrows for damaged and undamaged DNA, respectively.

In summary, we propose that Rad4’s lesion recognition relies on ‘kinetic gating’ mechanism to achieve selectively higher probability of ‘opening’ damaged DNA than normal DNA. In this mechanism, lesion recognition by Rad4/XPC leading to the ‘open’ conformation is controlled (‘gated’) by the competition between two kinetic parameters, as illustrated in Fig. 8: (1) opening time to form the thermodynamically stable, ‘open’ or recognition complex and (2) residence time per site as determined by the one-dimensional diffusion such as sliding and hopping of protein on DNA when it is in the loosely bound, nonspecific ‘search’ mode^33^. The opening rate for damaged DNA (red arrows) is expected to be larger than the opening rate for undamaged DNA (blue arrows), because of, for instance, the weakened base stacking and hydrogen bonding within a lesion. The residence time, on the other hand, may well be shorter for undamaged than for damaged DNA. The balance between these two factors together results in a higher net probability of Rad4/XPC opening damaged rather than undamaged DNA. This difference in the opening probabilities forms the basis for lesion recognition despite little difference in the thermodynamically most stable structures.

How proteins find their specific targets with a large number of closely related nonspecific molecules around is not limited to DNA-binding proteins, but is prevalent in biology. It is inevitable that erroneous bindings occur, but most systems are tolerant of such thermodynamic noise owing to intermediate ‘proofreading’ before proceeding down a pathway. ‘Kinetic proofreading’ originally proposed by Hopfield^38^ and Ninio^39^ is one such mechanism that detects and aborts incorrect bindings using chemical energy (for example, ATP hydrolysis). Our model showcases, for the first time, that ‘kinetic proofreading’ can be accomplished with neither chemical energy consumption nor difference in the thermodynamically most preferred states of the protein–DNA complexes^40^. During NER, lesion recognition by XPC is also followed by a bona fide, ATP-dependent damage verification by TFIIH, which further augments NER accuracy^3^. Finally, the DNA ‘opening’ induced by prolonged residence time may help explain the mechanisms of XPC within and beyond NER that cannot be explained solely by lesion-binding preferences of XPC^14^,^15^,^16^,^17^. In these cases, XPC-interacting proteins (for example, UV-damaged DNA-binding protein complex or DNA glycosylases) and/or post-translational modifications on XPC can help ‘stall’ the protein on DNA and induce opening of otherwise non-cognizant DNA. We also posit that ‘kinetic gating’ is a general target-recognition mechanism even for DNA-binding proteins that ultimately rely on direct structural discrimination, as a way of reducing wasteful interrogation at each and every site. Our results may have broad implications in genome maintenance, gene regulation, and cancer biology.

## Methods

### Oligonucleotide synthesis

Oligonucleotides containing disulphide-tethered cytosine were prepared by incorporating the 2-F-dI-CE phosphoramidite (Glen Research) at the desired position during solid-phase synthesis. The conversion and deprotection of 2-F-dI were performed according to Technical Bulletin provided by Glen Research (http://www.glenresearch.com/Technical/TB_2-F-dI.pdf). Briefly, 2-F-dI-containing oligonucleotides were reacted with cystamine to tether the disulphide group, then deprotected with 1,8-diazabicycloundec-7-ene. All synthetic oligonucleotides were purified with denaturing polyacrylamide gel electrophoresis.

### Preparation of Rad4–Rad23 complex

All Rad4–Rad23 complexes used in the study were overexpressed and purified from insect cells using previously described methods^8^. Briefly, the Hi5 insect cells co-expressing the Rad4–Rad23 complex were harvested 2 days after infection. After lysis, the proteins were purified using His-Select Nickel agarose resin (Sigma) and anion-exchange chromatography (Source Q, GE healthcare), followed by thrombin digestion and cation exchange (Source S, GE healthcare) and gel-filtration (Superdex200, GE healthcare) chromatography. Final sample was concentrated by ultrafiltration to ~13 mg ml^−1^ in 5 mM bis-tris propane–HCl (BTP-HCl), 800 mM NaCl and 5 mM dithiothreitol (DTT), pH 6.8.

To site-specifically tether Rad4 on DNA using disulphide crosslink, a V131C/C132S double mutation in Rad4 was introduced in the previously crystallized construct^8^ of the Rad4–Rad23 complex by site-directed mutagenesis. The V131C and C132S mutations were necessary and sufficient for efficient crosslinking of the purified Rad4–Rad23 complex with the DNA. The presence of seven other cysteines (C276, C354, C355, C463, C466, C509 and C572) of which the side chains were exposed on the surface of Rad4 did not affect the crosslinking, further signifying the specificity of the DNA binding and crosslinking. In fact, mutating these other cysteine residues to serines decreased the solubility of the protein and did not increase the crosslinking yield with DNA (data not shown).

The β-hairpin mutants of Rad4 used for T-jump experiments and electrophoretic mobility shift assays were prepared similarly. The mutant, ΔBHD3, lacked the entire BHD3 domain (residues 541–632, red in Fig. 1b,c), but instead had three extra glutamate residues (EEE) after residue 540 to increase the solubility of the protein. The other mutant, Δβ-hairpin3, lacked only the long β-hairpin region (residues 599–605, blue in Fig. 1b,c) in the BHD3. These mutations of Rad4 were incorporated in the context of the truncated Rad4–Rad23 complex previously described^8^.

### Disulphide crosslinking of Rad4–Rad23 complex with double-stranded DNA

To crosslink the protein and DNA, DTT in the Rad4–Rad23 storage buffer was first removed from the purified Rad4–Rad23 complex through a desalting column (Zeba Spin Desalting Column, 40,000 Da molecular weight cut-off, Thermo Scientific) pre-equilibrated in 5 mM BTP-HCl and 800 mM sodium chloride (NaCl), pH 6.8. The protein complex was subsequently mixed with DNA containing disulphide-modified base (G*, Fig. 1a,c) at 1:1 molar ratio at a final concentration of ~20 μM in crosslinking buffer (5 mM BTP-HCl, 100 mM NaCl and 10% glycerol, pH 6.8) at 4 °C overnight. The extent of the reaction was determined by SDS–PAGE under non-reducing conditions after treating the sample with 0.1 mM S-methylmethanethiosulfonate (Sigma) to quench the reaction. Typical crosslinking yield was ~50–70% (Supplementary Fig. 2).

### Purification of Rad4–Rad23–DNA complex

To remove unreacted protein and DNA, the crosslink reaction mixture was subject to anion-exchange chromatography (Mono Q, GE healthcare) over a 0–2 M NaCl gradient in 5 mM BTP-HCl and 10% glycerol, pH 6.8. The buffers were degassed by purging nitrogen. Purified Rad4–Rad23–DNA eluted at 400–480 mM NaCl and was further concentrated by ultrafiltration (Amicon, Millipore) to ~30 μM or 3 mg ml^−1^ (Supplementary Fig. 2).

### Crystallization, structure determination and refinement

All crystals were grown by the hanging-drop vapour diffusion method at 4 °C, mixing 1 μl of protein solution and 1 μl of crystallization buffer. Crystals of the complex appeared after a few days at 4 °C in wells containing 50 mM BTP-HCl, 100 mM NaCl, 12–16% isopropanol and 100 mM calcium chloride (CaCl_2_), pH 6.8. The crystals were then harvested with a harvest buffer using 20–30% polyethylene glycol (PEG) 200 or PEG400 as cryoprotectants, and were subsequently flash frozen in liquid nitrogen (Supplementary Fig. 2). Diffraction data were collected at −170 °C and were processed with the HKL3000 suite^41^. The structure of the Rad4–Rad23–DNA complex was determined by molecular replacement method using the previous structure (PDB code 2QSH, chains A and X containing only Rad4 and Rad23) as the search model and refined through multiple rounds of refinement in Phenix^42^. The final model contains residues 126–514 and 525–632 of Rad4 and 256–308 of Rad23. Figures are generated by Pymol (the PyMOL Molecular Graphics System, Version 1.2r3pre, Schrödinger, LLC).

### AFM studies of Rad4–DNA binding

The 514-bp undamaged DNA substrate was made by PCR from pSCW01 plasmid using the forward primer 5′- GCATTGCTGAGGGTTATTGTC -3′ and reverse primer 5′- TATCCGGTAAGCGGCAGG -3′. The PCR products were purified with QIAquick PCR purification kit (QIAGEN) and eluted in filtered deionized water.

To obtain AFM images, the purified Rad4–Rad23 complex used for crystallization (2 μM) was incubated with the 514-bp undamaged DNA substrate (400 nM) in binding buffer of 5 mM BTP-HCl, 160 mM NaCl, 5% glycerol and 0.74 mM 3-[(3-cholamidopropyl) dimethylammonio]-1-propanesulfonate (CHAPS), pH 6.8. Reaction mixtures were incubated at room temperature for 20 min and diluted by 1:80 in AFM deposition buffer of 25 mM sodium acetate, 25 mM 4-(2-hydroxyethyl)-1-piperazineethanesulfonic acid–potassium hydroxide (HEPES-KOH, pH 7.5) and 10 mM magnesium acetate. Ten μl of the dilution was deposited onto freshly cleaved mica, rinsed with deionized water and dried under a gentle stream of nitrogen gas. Images were collected by a MultiModeV microscope (Bruker Corporation) in an E scanner in tapping mode. Images were captured at a scan size of 1 μm × 1 μm, scan rate of ~3 Hz and resolution of 512 × 512 pixels. DNA contour lengths and bend angles from the obtained AFM images were measured using ImageJ. The bend angle data were binned into histograms and fitted to a Gaussian distribution using MATLAB.

### Fluorescence measurements and T-jump spectroscopy

Fluorescence measurements were carried out with DNA sequences containing a 3-bp mismatch (AN3) or 2-bp mismatch (AN21) in two different sequence contexts (Supplementary Table 1), with 2AP substituted for adenine at positions denoted by X. Measurements were also carried out with undamaged (matched) DNA construct (AN4; Supplementary Table 1). The 2AP-labelled DNA oligonucleotides were obtained from Oligos Etc. (Wilsonville, OR). All DNA oligonucleotides were purchased with high-performance liquid chromatography purification. All fluorescence measurements were done in phosphate-buffered saline buffer (10 mM disodium hydrogen phosphate (Na_2_HPO_4_), 2 mM monopotassium phosphate (KH_2_PO_4_), 137 mM NaCl and 2.7 mM potassium chloride (KCl), pH 7.4) with 1 mM DTT. The steady-state fluorescence emission spectra on 2AP-labelled DNA constructs, with and without bound Rad4, were measured on a FluoroMax4 spectrofluorimeter (Horiba Scientific, NJ). 2AP-labelled DNA duplexes were excited at 314 nm and emission spectra collected over the wavelength range 330–450 nm and temperature range 10–40 °C. Sample reversibility after the heating/cooling cycle was verified in equilibrium measurements on all samples, by comparing the fluorescence emission spectra measured at 25 °C before and after the heating/cooling cycle. For all DNA-only and protein–DNA complexes, with the exception of the complex with Δβ-hairpin3 mutant, reversible behaviour was observed with heating up to at least 40 °C. For Δβ-hairpin3–DNA complexes, however, irreversible behaviour was observed when the samples were heated to above 30 °C; therefore for measurements reported on this complex, the temperature range did not exceed 30 °C. The concentrations for the equilibrium measurements were 10 μM DNA each with 1:1 ratio of protein:DNA for all complexes. The intensities of all the fluorescence emission spectra shown are scaled relative to the spectra of 3-bp mismatch DNA-only sample (AN3), which was normalized such that the maximum of the spectrum measured at 25 °C was 1 (in arbitrary units).

The kinetics measurements were performed using a home-built laser T-jump spectrometer, as described previously^43^. Briefly, 10 ns laser pulses at 1,550 nm, generated by Raman shifting the 1,064 nm pulses from the output of an Nd:YAG laser, are focused to ~1 mm spot size on to a sample cuvette of path length 0.5 mm, which yields ~10 °C T-jump at the centre of the heated volume. The magnitude of the T-jump was determined by measurements on control samples (2AP-labelled DNA only) by a comparison of the intensity observed in the kinetics experiments immediately after the T-jump with the intensity observed in the equilibrium measurements of the temperature dependence of the 2AP fluorescence for these samples. The errors in the T-jump estimates are about 10–20%. The probe source for excitation of 2AP fluorescence was a 200-W Hg–Xe lamp, with the excitation wavelengths selected by a broadband filter with transmission in the range of 300–330 nm. The probe light was focused on a ~300-μm spot in the middle of the heated volume. The fluorescence emission intensity was monitored perpendicular to the excitation direction, with a combination of a long-pass filter (>352 nm) and a short-pass filter (388 nm), and measured with a Hamamatsu R928 photomultiplier tube and a 500-MHz transient digitizer. The protein and DNA concentrations for the T-jump measurements were 60 μM each.

## Author contributions

X.C. carried out protein engineering and purifications, protein–DNA crosslinking and crystallization experiments with contributions from B.P. and Y.S. G.Z. and C.H. synthesized modified oligonucleotides for crosslinking experiments. X.C., Y.K. and J.-H.M. collected and analysed crystallographic data. J.-H.M. and Y.K. did model building and refinement. Y.V. and A.A. designed the fluorescence measurements on complexes with 2AP-labelled DNA substrates with contributions from X.C. and J.-H.M. Y.V. carried out the equilibrium and T-jump experiments and analysed the relaxation traces to obtain the DNA-opening times. L.L. and B.V.H. carried out the AFM studies. A.A. and J.-H.M. wrote the manuscript with contributions from all authors.

## Additional information

**How to cite this article**: Chen, X. *et al*. Kinetic gating mechanism of DNA damage recognition by Rad4/XPC. *Nat. Commun.* 6:5849 doi: 10.1038/ncomms6849 (2015).

**Accession codes:** The structural factor have been deposited with the protein data bank (PDB) under accession number 4U29.

## Supplementary Material

Supplementary Information Supplementary Figures 1-5, Supplementary Tables 1-2, Supplementary Methods and Supplementary References

## Figures and Tables

**Table 1:** Data collection and refinement statistics.

	**xc149**
*Data collection*	
Space group	P 4_1_ 2_1_ 2
Cell dimensions	
*a, b, c* (Å)	79.405, 79.405, 404.366
*α*, *β*, *γ* (°)	90, 90, 90
Resolution (Å)	50.00–3.05 (3.10–3.05)
*R*_sym_ or *R*_merge_	7.1% (79.2%)
*I*/*σI*	24.56 (2.3)
Completeness (%)	99.9% (100%)
Redundancy	6.7 (6.9)
	
*Refinement*	
Resolution (Å)	39.7–3.05
No. of reflections	25,974
*R*_work_/*R*_free_ (%)	20.41/25.66
No. of atoms	5,447
Protein	4,508
DNA	939
Water	0
*B*-factors (Å^2^)	68.00
Protein	63.30
DNA (G47)	90.60 (91.67)
Water	0
r.m.s. deviations	
Bond lengths (Å)	0.017
Bond angles (°)	1.94

r.m.s., root mean squared.

Values in parentheses are for highest-resolution shell.
