# Supplementary material for: Kinetic gating mechanism of DNA damage recognition by Rad4/XPC
Source: Nat Commun. 2015 Jan 6;6:5849. doi: 10.1038/ncomms6849 (PMC4354021; doi:10.1038/ncomms6849)
Supplement: Supplementary Information — Supplementary Figures 1-5, Supplementary Tables 1-2, Supplementary Methods and Supplementary References [file ncomms6849-s1.pdf]

SUPPLEMENTARY INFORMATION (FIGURES, TABLES & METHODS)

a

Mismatch DNA:
5′-TTGACTCGACATCC**CCC**GCTACAA -3′
3′- ACTGAGCTGTAGG**CCC**CGATGTTA -5′

Undamaged DNA:
5′-TTGACTCGACATCC**CCC**GCTACAA -3′
3′- ACTGAGCTGTAGG**GGG**CGATGTTA -5′

b

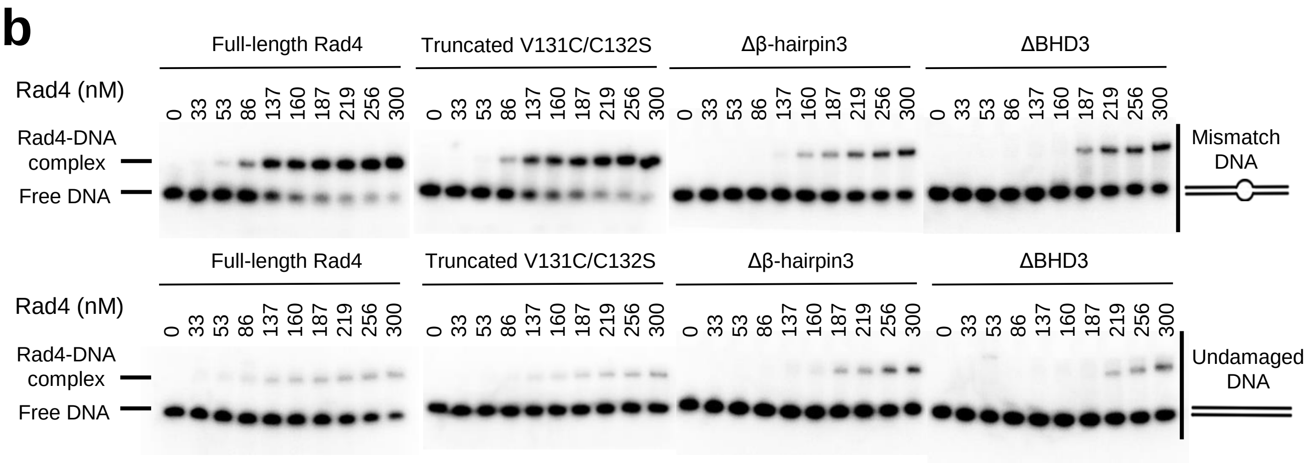

c

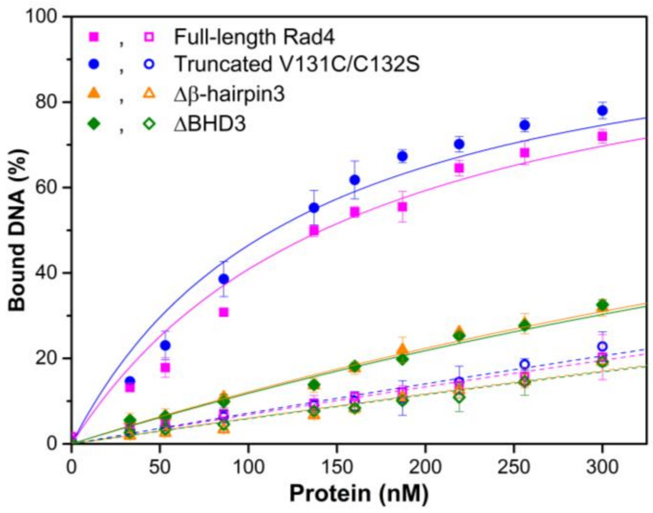

	Full-length Rad4	Truncated V131C/C132S	Δβ-hairpin3	ΔBHD3
K_s (nM)	45 ± 4	31 ± 3	280 ± 8	293 ± 8
R^2	0.954	0.958	0.987	0.986
K_{ns} (nM)	430 ± 45	363 ± 45	626 ± 57	650 ± 56
R^2	0.956	0.964	0.967	0.965

Supplementary Figure 1. Characterization of DNA-binding activities of the Rad4 constructs.

To check whether the double mutations V131C/C132S introduced for crosslinking affects the intrinsic DNA binding of the Rad4–Rad23 complex used for crystallization, the DNA binding of this mutant was tested and compared with the full-length Rad4–Rad23 complex using electrophoretic mobility shift assays (EMSA) (Supplementary Methods, ref. 1). The DNA-binding activities of the β-hairpin mutants used in T-jump studies (ΔBHD3 and Δβ-hairpin3) were also tested and compared. While the full-length Rad4–Rad23 complex contained full-length Rad23, all other Rad4 constructs were expressed with Rad23 truncated as described[1].

a. The sequences of 24-bp damaged/mismatch and normal duplex DNA constructs used for the assay. Note the undamaged DNA sequence is the same as that used for crystallization (**Fig. 1c**, *bottom*) except that G8 (marked red) is an unmodified guanosine. The nucleotides shown in bold are those that differ in the mismatch and undamaged DNA sequences. The boxed nucleotides show the positions of those that would get flipped out when specifically bound to Rad4 (mismatch DNA) or when tethered to Rad4 (undamaged DNA).

b. Typical gel images showing the Rad4 constructs binding to mismatch DNA (*top*) and undamaged DNA (*bottom*).

c. Quantification of the percent bound DNA fractions in **b** versus concentration of protein. The symbols and error bars indicate the means and ranges as calculated by ± sample standard deviation, respectively, from triplicate gel shift experiments. K_d values for specific binding to mismatch DNA (K_s) and for nonspecific binding for undamaged DNA (K_{ns}) were obtained as described[1]. Solid lines indicate the fit curves of the data points of the same color. Note that the presence of single 5'-T/A overhangs in the DNA duplexes used in this study did not significantly affect the K_s or K_{ns} compared with those of analogous DNA constructs with blunt-ends used previously[1], also consistent with other studies[2].

The data show that V131C/C132S mutations did not affect DNA binding of Rad4. The ΔBHD3 and Δβ-hairpin3 mutants, however, exhibit significantly weakened binding to mismatch DNA while retaining affinities to undamaged DNA, resulting in significant loss in lesion recognition specificity.

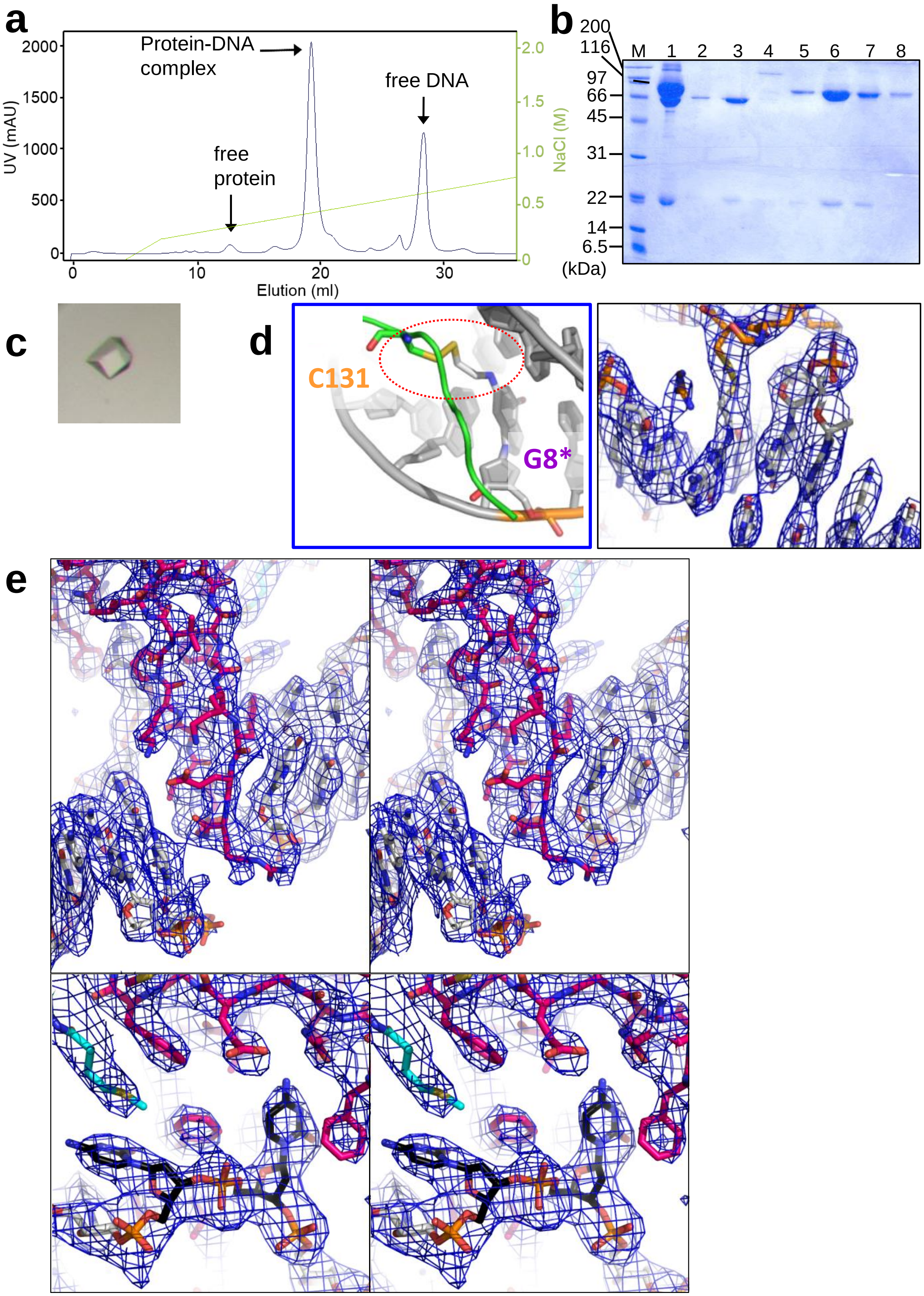

Supplementary Figure 2. Purification and crystallization of Rad4–Rad23 complex

crosslinked to undamaged double-stranded DNA.

a. The crosslinked Rad4–Rad23–DNA complex was purified over a MonoQ column (GE Healthcare) with 0–2 M NaCl gradient. The separated peaks corresponding to the crosslinked complex and the free protein and DNA are indicated with arrows.

b. Non-reducing SDS-PAGE gels show that the crosslinked complex (Lane 5,6,7) were separated from the free proteins (Lane 2, 3). To first quench the crosslinking reaction, all the samples were mixed with 0.1 mM of S-methyl methanethiosulfonate (Sigma) and incubated at room temperature for 10 min. Subsequently the samples were loaded to 15% SDS-PAGE gel using a loading buffer lacking 2-mercaptoethanol. Gels were run at 180 V for 50 min. Lane 1 contains crosslinking reaction mixture before purification; lane 2 and 3 shows free protein band eluting at 280–320 mM NaCl; lane 5-8 shows the crosslinked complex eluting at 400–480 mM NaCl.

c. Crystal photo of the Rad4–Rad23 complex crosslinked to 24-bp undamaged duplex DNA (**Fig. 1**). The crystal was grown in 5 mM BTP-HCl, 200 mM NaCl, 15.5% isopropanol, 100 mM $CaCl_2$, pH 6.8.

d. (*left*) Close-up view of the disulphide tether formed between Rad4 and DNA. The disulphide bond between the C131 of Rad4 and G*8 of the DNA is circled in red dotted lines. (*right*) 2*Fo-Fc* map of the region near the crosslink at a contour level of 2σ.

e. (*left*) Stereo electron density map showing the β-hairpin from BHD3 domain penetrating into DNA duplex and (*right*) a stereo map near the flipped-out cytosines 16 and 17 shown in **Fig. 1c**. The 2*Fo-Fc* maps are shown at a contour level of 1σ.

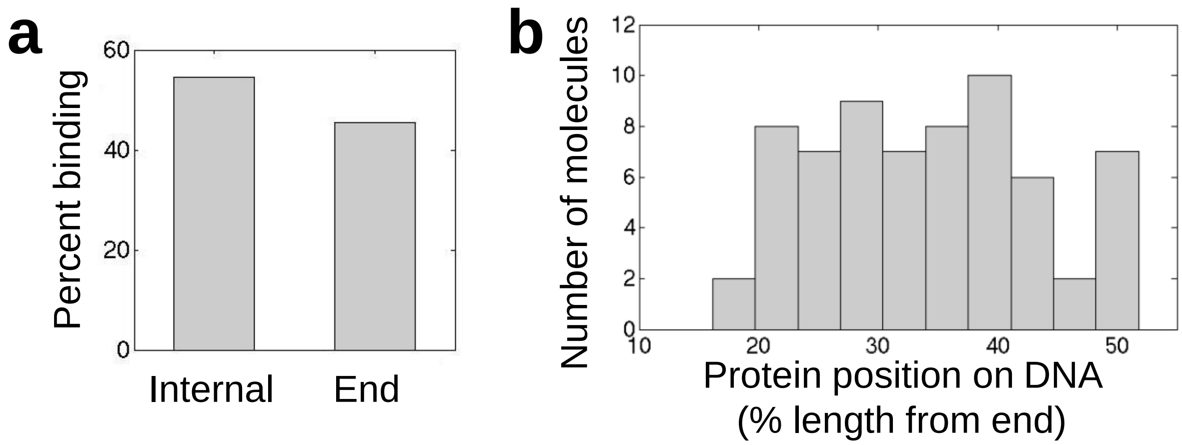

Supplementary Figure 3. AFM measurements of Rad4 bound to undamaged DNA.

a. Plot of end versus non-specific, internal binding of Rad4 on 514-bp undamaged DNA. Among 119 DNA binding molecules, 55% were internal binding and 45% were end-binding.

b. Histogram of the internal binding position of Rad4 along DNA. The positions on DNA were measured as the shortest distance from protein to DNA end and calculated by percent of total DNA length. A total of 67 molecules were measured. The apparently low frequency of Rad4 binding near the end of DNA (10–20% position) is due to the exclusion of molecules with protein bound at an end as well as to the resolution limit that hampers the detection of very short segments of DNA extending beyond a bound protein. Aside from the end-region of the DNA, however, the distribution of Rad4 is mostly random throughout the rest of the DNA segment (20–50% position). The only exception seems to be at position ~ 47%, which appears to have less Rad4 bound than at other internal regions. There could be multiple reasons why internally bound Rad4 may exhibit certain degree of non-randomness. For instance, it may be due to sequence-dependent preference of Rad4 for certain stretches of DNA versus others. Such preference may arise from any propensity of the DNA sequence to be more easily bent, unwound, and/or opened up by Rad4 to form the “open” conformation.

Supplementary Figure 4. Sequence-context dependence of Rad4-induced DNA-opening dynamics. 2AP fluorescence measurements similar to those reported for AN3 (**Fig. 4**) were carried out for another mismatched sequence, AN21 (**Supplementary Table 1**). AN21 contains a 2-bp mismatch and more closely resembles the sequence used in crystallographic studies.

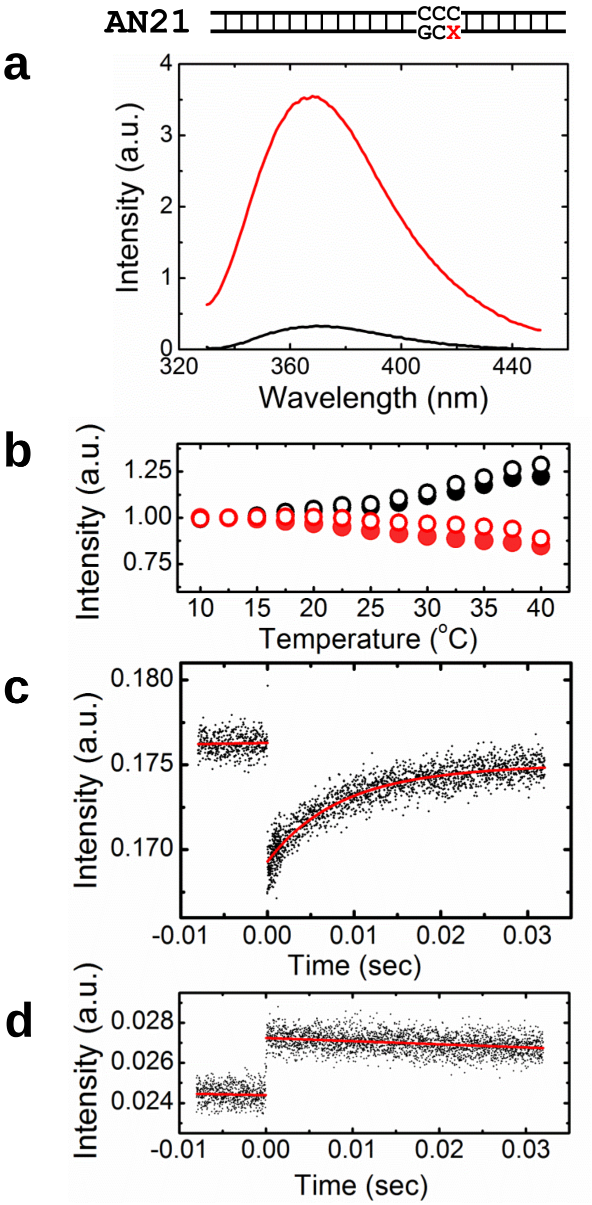

a. The 2AP fluorescence emission spectra measured for DNA alone (black) and Rad4–DNA complex (red), with excitation at 314 nm at 25 °C. The fluorescence emission intensities for AN21 increased ~10-fold upon Rad4 binding.

b. The maxima of the 2AP fluorescence emission spectra, measured at 365 nm as a function of temperature, for DNA alone (black) and Rad4–DNA complex (red); the intensities are normalized to match at the lowest temperature. Open and filled symbols are for two independent sets of measurements on each sample. Notably, 2AP fluorescence in AN21 alone increases with increasing temperature, in contrast to the behavior observed in AN3, which showed a decrease in 2AP fluorescence with increasing temperature (**Fig. 4b**, *left*). Thus, in the AN21 context, 2AP in free DNA has an increased propensity to unstack as the temperature is raised.

c. Relaxation traces measured on the Rad4–DNA complex, in response to a 4 ºC T-jump, show single-exponential kinetics, with relaxation time 8.0 ± 1.9 ms (at final temperature 21 ºC). The relaxation rates measured on the Rad4–AN21 and Rad4–AN3 complexes overlap (**Fig. 6**), indicating that the rate-limiting step in forming the "open" conformation is not sequence-context dependent.

d. Relaxation kinetics measured on the AN21 DNA-only sample exhibited much slower kinetics, with relaxation time 169 ± 98 ms, similar to T-jump recovery kinetics back to the initial temperature. The uncertainty is the sample standard deviation obtained from two independent measurements. Note that the intensity immediately after the T-jump increases for this sample, consistent with its equilibrium temperature-dependence (panel **b**), indicating that 2AP unstacking is too fast to be resolved on the ~5 μs time-resolution of our T-jump instrument. In contrast, for the Rad4-AN21 complex, the intensity immediately after T-jump drops (panel **c**), indicating that rapid 2AP unstacking is suppressed in the complex.

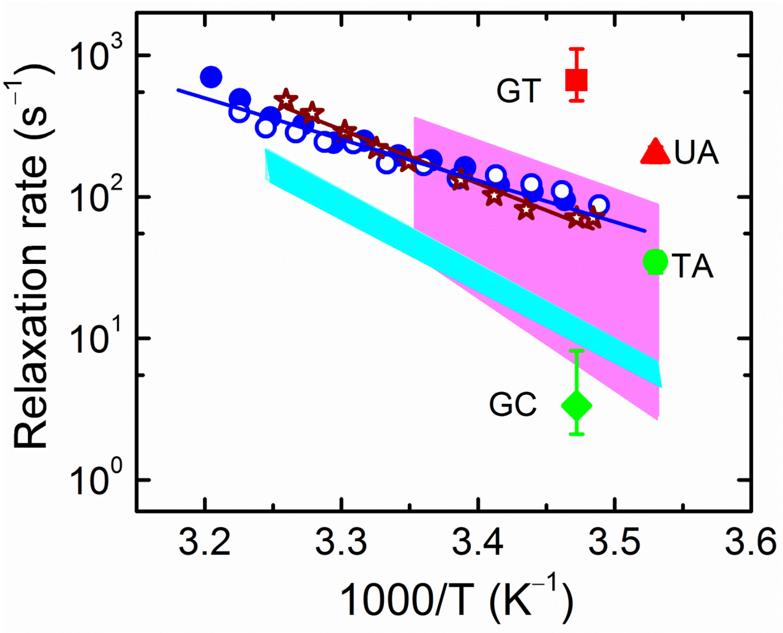

Supplementary Figure 5. DNA opening dynamics in full-length Rad4 in complex with mismatch DNA. Relaxation rates obtained from T-jump measurements carried out with full-length Rad4 bound to mismatch DNA substrate (AN3; brown stars) are plotted versus the inverse of the final temperature (after the T-jump). The blue circles are the relaxation rates measured with the truncated Rad4 construct[1] as shown in **Fig. 6**, with the open and filled circles indicating two independent sets of measurements. The continuous (brown and blue) lines are an Arrhenius fit to the relaxation rates for each sample.

The pink (cyan) shaded regions represent the range of base-pair opening ("breathing") rates for A/T (G/C) base pairs, from NMR imino proton exchange measurements of Coman and Russu[3]. The opening rates and their ranges are also indicated for G/T mismatch (red square)[4] and U/A mismatch (red triangle)[5], T/A base pair (green circle)[5] and G/C base pair (green diamond) [4]. These descriptions are the same as in the legend to **Fig. 6**.

Supplementary Table 1. DNA sequences used for T-jump studies.

Name	Sequence
AN3	5'-CGTGACTCAACATCC**TAT**GCTACAACTCAGTGC - 3'<br>3'-GCACTGAGTTGTAGG**TXT**CGATGTTGAGTCACG - 5'
AN4	5'-CGTGACTCAACATCC**ATA**GCTACAACTCAGTGC - 3'<br>3'-GCACTGAGTTGTAGG**TXT**CGATGTTGAGTCACG - 5'
AN21*	5′-TTGACTCGACATCCC**CC**GCTACAA -3′<br>3′- ACTGAGCTGTAGGG**CX**CGATGTTA -5′

X denotes 2-aminopurine (2AP). Red indicates mismatched base pairs; green indicates the corresponding matched ones.

* The length and sequence of AN21 was chosen to match those used for structural studies (ref. 1 & **Fig. 1c**). Note that AN21 has a 2-bp mismatch rather than a 3-bp mismatch as in AN3.

Supplementary Table 2. Diffusion constants (D) and residence times per base of various DNA-repair proteins on normal DNA duplexes.

Protein	D ($m^2 s^{-1}$)	D ($bp^2 s^{-1}$)	Residence time (μs)
hUNG[6]		6 $x10^3$	83
hOGG1[7]	6 $x10^{-13}$ □□	5 $x10^6$	0.1
MutM[7]	5 $x10^{-14}$ □□	4 $x10^5$	1.4
Msh2-Msh6[8]	9 $x10^{-14}$ □<br>– 2 $x10^{-16}$ □	8 $x10^5$<br>– 2 $x10^3$	0.6<br>– 289

Supplementary Methods

Electrophoretic mobility shift assay (EMSA) The Rad4-Rad23 constructs were purified in the same way as the complex used for crystallization (see Methods), but were not cleaved by thrombin. For binding, the protein complex was mixed with either 1 nM ^{32}P-labelled 24-bp undamaged/matched duplex DNA or damaged/mismatch DNA in the presence of 1000 nM cold, matched DNA in a buffer of 5 mM BTP-HCl, 75 mM NaCl, 5 mM DTT, 5% glycerol, 0.74 mM 3-[(3-cholamidopropyl)dimethylammonio]-1-propanesulfonate (CHAPS), 500 μg ml^{-1} bovine serum albumin, pH 6.8. The sequences for the matched and mismatch DNA duplexes used in the assay are shown in **Supplementary Fig. 1a.** The binding reactions were incubated at room temperature for 20 min, and were subsequently separated on 4.8% non-denaturing polyacrylamide gels in 1x TBE buffer for 15–18 min at 4 °C. The gels were quantitated by autoradiography using Personal Molecular Imager™ and Quantity One software (Bio-Rad) using equations described in ref. 1 .

Supplementary References

1. Min JH, Pavletich NP. Recognition of DNA damage by the Rad4 nucleotide excision repair protein. *Nature* **449**, 570-575 (2007).

2. Sugasawa K, Shimizu Y, Iwai S, Hanaoka F. A molecular mechanism for DNA damage recognition by the xeroderma pigmentosum group C protein complex. *DNA Repair* **1**, 95-107 (2002).

3. Coman D, Russu IM. A nuclear magnetic resonance investigation of the energetics of basepair opening pathways in DNA. *Biophys J* **89**, 3285-3292 (2005).

4. Moe JG, Russu IM. Kinetics and energetics of base-pair opening in 5'-d(CGCGAATTCGCG)-3' and a substituted dodecamer containing G.T mismatches. *Biochemistry (Mosc)* **31**, 8421-8428 (1992).

5. Parker JB, Bianchet MA, Krosky DJ, Friedman JI, Amzel LM, Stivers JT. Enzymatic capture of an extrahelical thymine in the search for uracil in DNA. *Nature* **449**, 433-437 (2007).

6. Schonhoft JD, Stivers JT. Timing facilitated site transfer of an enzyme on DNA. *Nat Chem Biol* **8**, 205-210 (2012).

7. Blainey PC, van Oijen AM, Banerjee A, Verdine GL, Xie XS. A base-excision DNA-repair protein finds intrahelical lesion bases by fast sliding in contact with DNA. *Proc Natl Acad Sci U S A* **103**, 5752-5757 (2006).

8. Gorman J*, et al.* Dynamic basis for one-dimensional DNA scanning by the mismatch repair complex Msh2-Msh6. *Mol Cell* **28***,* 359-370 (2007).
